# Juvenile polyposis syndrome: A case report

**DOI:** 10.1002/ccr3.6798

**Published:** 2023-01-03

**Authors:** Edwin Mogere, Elijah Mwaura, Mark Waithaka, Victor Mutua, Maurice Mugao, Chris von Csefalvay, Dennis Mukamati

**Affiliations:** ^1^ Murang'a Level 5 Hospital Murang'a Kenya; ^2^ P.C.E.A Chogoria Mission Hospital Tharaka Nithi Kenya; ^3^ Starschema Inc. Office of Intramural Research Arlington Virginia USA

**Keywords:** colon cancer, gastroenterology, juvenile polyposis syndrome

## Abstract

Juvenile polyposis syndrome (JPS) is an autosomal dominant disease that is characterized by multiple hamartomatous polyps. Patients with JPS are at increased risk for developing colorectal and gastric cancer. JPS was diagnosed by endoscopy and histology, and the patient underwent surgery, total proctocolectomy and ileal pouch–anal anastomosis.

## Abstract 2

“JPS ought to be suspected in patients with multiple‐stalked polyps. Endoscopic studies may not be sufficient to pinpoint the diagnosis but can be a useful guide in diagnosis of JPS. Histology with genetics can be used as confirmatory tests.”

## 1 INTRODUCTION

Juvenile polyposis syndrome (JPS) is an autosomal dominant disease that is characterized by multiple hamartomatous polyps throughout the gastrointestinal tract. JPS is rare, with an approximated incidence of 1 in 100,000 individuals.^1^, ^2^ Patients with JPS are at increased risk for developing GI malignancies such as colorectal and gastric cancer.^3^, ^4^ The cumulative risks of developing colorectal and gastric cancers are estimated to be approximately 40%–50% and 20%, respectively.^5^, ^6^, ^7^

Other hamatomatous polyposis syndromes include Peutz–Jeghers syndrome (PJS), Bannayan–Riley–Ruvalcaba syndrome, and Cowden syndrome.^8^ Unlike PJS, which is commonly characterized by small bowel polyps, in JPS polyps primarily occur in the stomach and colon.^6^, ^9^

JPS occurs due to germline mutations in the SMAD4 or bone morphogenetic protein receptor type‐1A (BMPR1A) genes, which are related to the transforming growth factor‐beta signaling pathway.^10^, ^11^

The diagnosis of polyposis requires endoscopy of both upper and lower gastrointestinal tract cytology. Herein, we describe an uncommon cause of lower GI bleeding in a 13‐year‐old boy, which necessitated surgical intervention.

## 2 CASE PRESENTATION

A 13‐year‐old boy who presented to our facility with a history of painless hematochezia for 3 years. He developed pallor and generalized body weakness over time. His family history was negative for diagnosed colonic polyps and cancer.

On physical examination, he looked unwell, and while afebrile, his blood pressure was 103/68 mmHg and pulse at 96 bpm. He exhibited moderate conjunctival pallor, but no jaundice and no koilonychia. The abdomen was not distended, soft on palpation with no areas of tenderness, and bowel sounds were present. Digital rectal examination revealed several soft polypoid masses located in the rectum, blood on examining finger, and normal anal tone.

The full hemogram test revealed a hypochromic microcytic anemia; hemoglobin of 8.4 g/dL, hematocrit of 29.4%, mean corpuscular volume of 55.8 fl and a mean corpuscular hemoglobin of 15.9 pg. The white blood cell count was normal at 4.8 × 10^9^/L. The other laboratory tests were within normal ranges.

**FIGURE 1:**
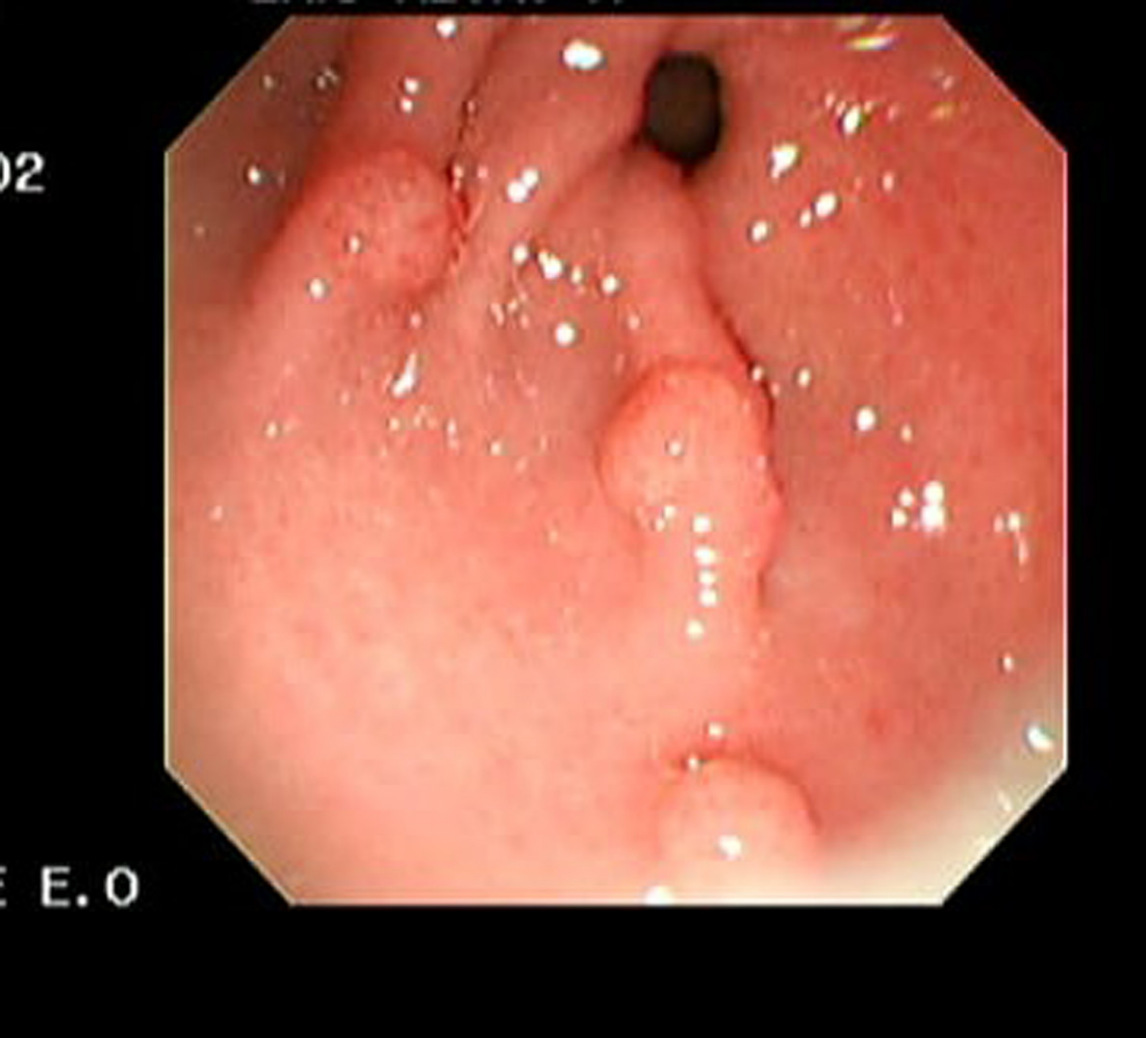
OGD showing 5 pedunculated gastric polyps

**FIGURE 2:**
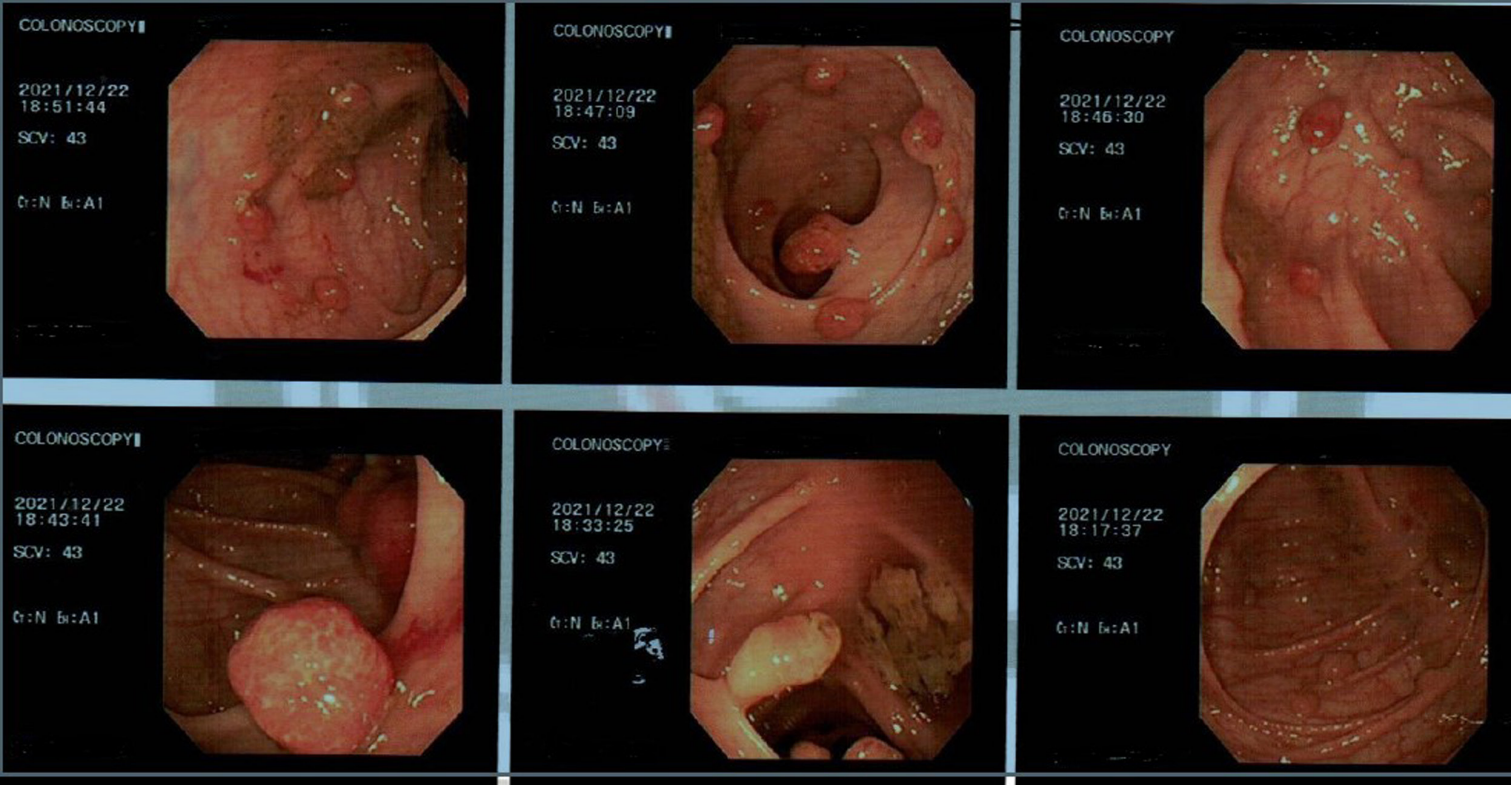
Colonoscopy illustrated multiple pedunculated polyps throughout the colon

Upper gastrointestinal tract endoscopy revealed 5 polyps (Figure 1), and gastric polyps biopsies were submitted for histopathology. Submitted fragments exhibited moderate chronic inflammatory cell infiltrate of lymphocytes, negative for H pylori, and there was no metaplasia nor dysplasia. Colonoscopy illustrated multiple pedunculated polyps throughout the colon (Figure 2). This substantiated a diagnosis of JPS.

**FIGURE 3:**
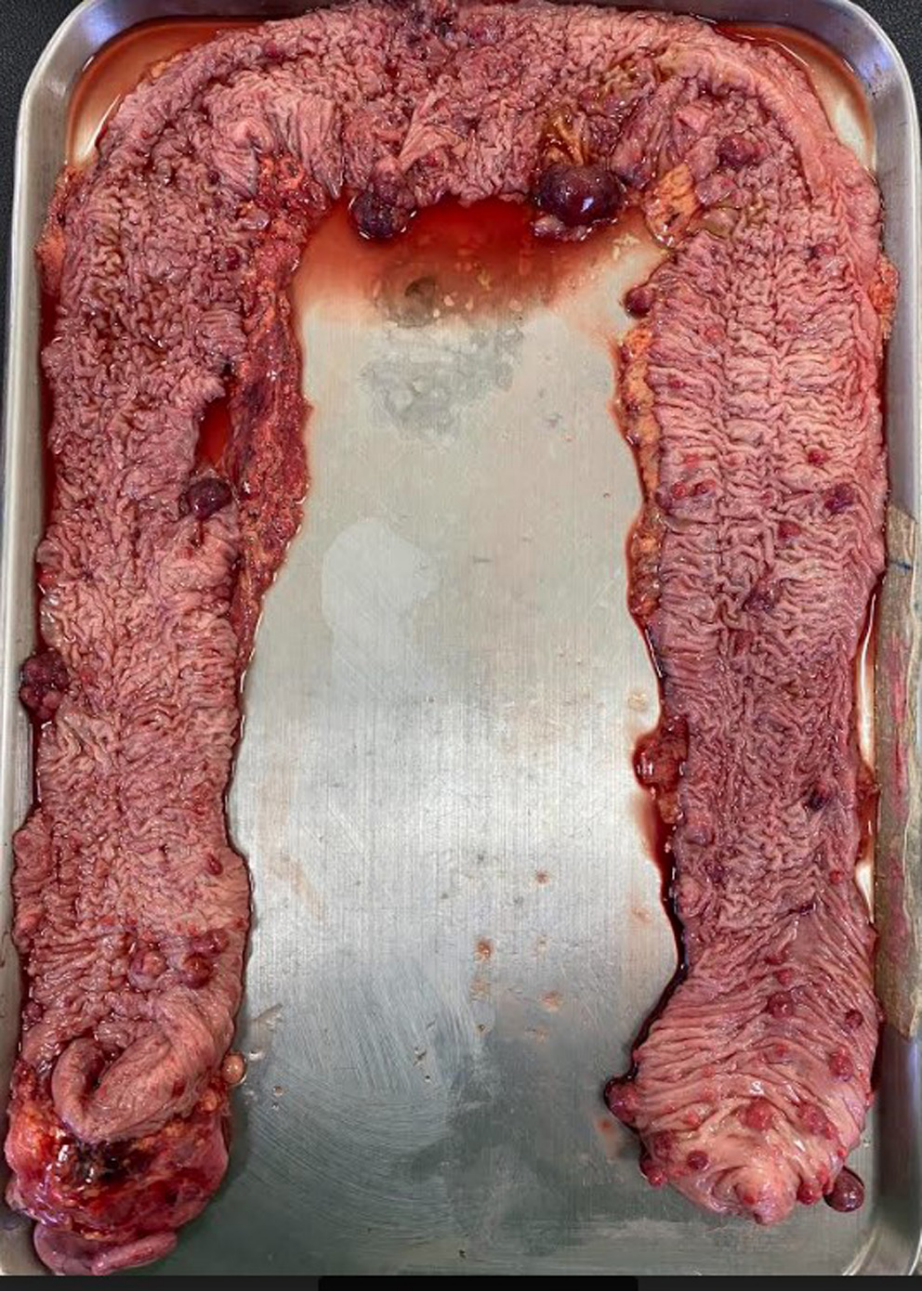
View of the resected specimen, revealing multiple polyps from the cecum to the rectum

Following the diagnosis of JPS with significant bleeding that lead to symptomatic anemia, total proctocolectomy and ileal pouch–anal anastomosis was performed. The abdomen was opened by an extended midline incision, and a thorough exploration was performed. The colon was mobilized by dissecting the white line of Toldt to the level of the rectum while protecting the ureters. The appropriate point of division of the terminal ileum was selected, and its transection with hemostasis was achieved with serial clamping, division, and ligation of vessels. Distal bowel transection was performed at the rectum, with careful dissection starting from posterior then anterior then laterally. A J‐pouch was created and anastomosed to the anal canal using a circular stapler and the patency of the anastomosis was confirmed, with no air leak. The abdomen was copiously irrigated with saline, and midline incision was closed in layers. Resected specimen was opened up and demonstrated 109 polyps (Figure 3). Histology of resected specimen reported findings consistent with JPS and negative for high‐grade dysplasia or malignancy.

The patient was discharged on day 3 post‐op, able to feed small meals and retaining. He complained of fecal incontinence for a few days, which resolved in 1 week. The patient was reviewed at our surgical clinic at 3‐month post‐op. He had gained weight and did not have any complaints other than opening bowels 4–6 times a day. Follow‐up endoscopies at 3 months revealed 1 polyp at the anorectal stump and 5 gastric polyps. The patient together with his guardian preferred the gastric polyps and the anorectal stump polyp to be resected after completion of his primary school education. He is currently in his final year.

## 3 DISCUSSION

Juvenile polyposis syndrome (JPS) is an autosomal dominant disease that is characterized by multiple hamartomatous polyps. According to Guillén‐Ponce et al., the diagnosis of JPS can be confirmed if patient has any one of three findings: (1) ≥5 polyps in the colon or rectum, (2) numerous juvenile polyps throughout the GI tract, or (3) any number of juvenile polyps with family history of JPS.^9^, ^12^, ^13^ In our report, the patient met two of these three findings.

The gross appearance of almost all polyps in patients with JPS is that of stalked polyps. Elitsar et al., a review of patients with JPS revealed that 90.5% were stalked and only 9.5% were sessile.^14^ Therefore, endoscopy studies could help to differentiate FAP (familial adenomatous polyposis) from JPS, as in FAP, most polys have been noted to be sessile.^15^

JPS has been reported to be a risk for colorectal and gastric cancer. Most polyps in JPS are benign hamartomatous polyps with no dysplasia. Viele et al. reviewed 767 colorectal JPS polyps and found 8.5% of the polyps contained mild‐to‐moderate dysplasia, and only 0.3% demonstrated severe dysplasia or cancer.^3^ Therefore, to reduce the risk of malignancy, all polyps in the GI tract of patients with JPS should be removed, either endoscopically or through bowel resection. Of note is patients with mild polyposis can be managed by frequent endoscopic resection with serial endoscopic review studies.

Current indications for surgery include the following: (1) extravagant polyp burden, (2) severe blood loss leading to persistent anemia, and (3) juvenile polyps showing features of severe dysplasia, malignant polyps, or if there is a strong family history of colorectal cancer. Our patient met two of the three indications for surgery. Surgical options for JPS include the following: (1) colectomy [segmental or subtotal colectomy or total colectomy (TC)] with ileal rectal anastomosis (IRA) and (2) total proctocolectomy (TPC) with ileal pouch–anal anastomosis (IPAA). In our case, the definitive treatment we opted for was proctocolectomy with ileoanal J‐pouch due to the presence of multiple polyps in the rectum and colon.

In conclusion, JPS ought to be suspected in patients with multiple‐stalked polyps. Endoscopic studies may not be sufficient to pinpoint the diagnosis, but can be a useful guide and differential diagnosis of JPS versus FAP can be inferred from endoscopic appearance and if available, histopathology and genetic investigations to help confirm the diagnosis.

## AUTHOR CONTRIBUTIONS

**Edwin Mogere:** Conceptualization; writing – original draft. **Elijah Mwaura:** Conceptualization; investigation; writing – review and editing. **Mark Mwangi:** Conceptualization; investigation; writing – review and editing. **victor Mutua:** Conceptualization; writing – original draft. **Maurice Mugao:** Supervision; writing – review and editing. **Chris von Csefalvay:** Supervision; writing – review and editing. **Dennis Mukamati:** Writing – review and editing.

## FUNDING INFORMATION

None.

## CONFLICT OF INTEREST

The authors declare no conflict of interest.

## CONSENT

Written informed consent was obtained from the patient to publish this report.

## Data Availability

Data available on request from the authors
